# Understanding and modelling the economic impact of spinal cord injuries in the United Kingdom

**DOI:** 10.1038/s41393-019-0285-1

**Published:** 2019-05-13

**Authors:** David McDaid, A-La Park, Angela Gall, Mariel Purcell, Mark Bacon

**Affiliations:** 1 Personal Social Services Research Unit, Department of Health Policy, London School of Economics and Political Science, London, UK; 2 London Spinal Cord Injury Centre, Royal National Orthopaedic Hospital, Stanmore, UK; 3 University College London, London, UK; 4 Queen Elizabeth National Spinal Injuries Unit, Queen Elizabeth University Hospital, Glasgow, UK; 5 Spinal Research, London, UK; 6 Present Address: Department of Rehabilitation Medicine, Woodend Hospital, Aberdeen and University of Aberdeen, Aberdeen, UK

**Keywords:** Health care economics, Health policy

## Abstract

**Study design:**

Economic modelling analysis.

**Objectives:**

To determine lifetime direct and indirect costs from initial hospitalisation of all expected new traumatic and non-traumatic spinal cord injuries (SCI) over 12 months.

**Setting:**

United Kingdom (UK).

**Methods:**

Incidence-based approach to assessing costs from a societal perspective, including immediate and ongoing health, rehabilitation and long-term care directly attributable to SCI, as well as aids and adaptations, unpaid informal care and participation in employment. The model accounts for differences in injury severity, gender, age at onset and life expectancy.

**Results:**

Lifetime costs for an expected 1270 new cases of SCI per annum conservatively estimated as £1.43 billion (2016 prices). This equates to a mean £1.12 million (median £0.72 million) per SCI case, ranging from £0.47 million (median £0.40 million) for an AIS grade D injury to £1.87 million (median £1.95 million) for tetraplegia AIS A–C grade injuries. Seventy-one percent of lifetime costs potentially are paid by the public purse with remaining costs due to reduced employment and carer time.

**Conclusions:**

Despite the magnitude of costs, and being comparable with international estimates, this first analysis of SCI costs in the UK is likely to be conservative. Findings are particularly sensitive to the level and costs of long-term home and residential care. The analysis demonstrates how modelling can be used to highlight economic impacts of SCI rapidly to policymakers, illustrate how changes in future patterns of injury influence costs and help inform future economic evaluations of actions to prevent and/or reduce the impact of SCIs.

## Introduction

The United Kingdom (UK) experiences around 16 new cases per million population in traumatic spinal cord injuries [1, 2] and 2–3 new cases per million population in non-traumatic spinal cord injuries per annum [1]. This is >1200 new spinal cord injuries (SCIs) every year, the majority due to traumatic events, with the remainder resulting from disease, such as non-malignant tumours.

These SCIs can have devastating, life-changing impacts on those injured and their families. Economic evaluation can help make the case for investing in actions to reduce these injuries, as well as better manage and support people living with SCI. It can help determine the incremental cost-effectiveness of actions compared to usual care.

A pre-requisite for any economic evaluation is to quantify the economic costs of SCI to different sectors/stakeholders. These include National Health Service (NHS) costs for immediate and ongoing healthcare use, not only in dealing with SCI and its many chronic complications, including pressure sores, respiratory, cardiovascular and urinary/bowel problems [3], but also treating higher risks of multi-morbidity, such as depression and obesity [4]. Social care services, the NHS and people with SCI will also share rehabilitation, residential and home care costs, including aids and modifications. Public employment and education services will fund reintegration into work or school. Indirect costs borne by society typically relate to lost employment and the need for family members to give up their time to provide informal care.

Despite the importance of economic evidence in health policymaking in the UK and many countries, there are surprisingly few estimates of SCI costs. While UK studies on the cost-effectiveness of interventions for SCI and complications have been published [5], no detailed estimates of overall costs of SCI to the healthcare system, wider public purse and society in a UK context exist. Indeed, National Institute for Health and Care Excellence (NICE) guidelines relied on an expert opinion estimate of £2.5 million in lifetime costs for spinal injury, with values ranging from £0 to £10 million [2]. Given this lack of information, we aimed to model lifetime costs of SCI in the UK. This will also have broader value, as the model could be adapted to other country contexts.

## Methods

**Fig. 1:**
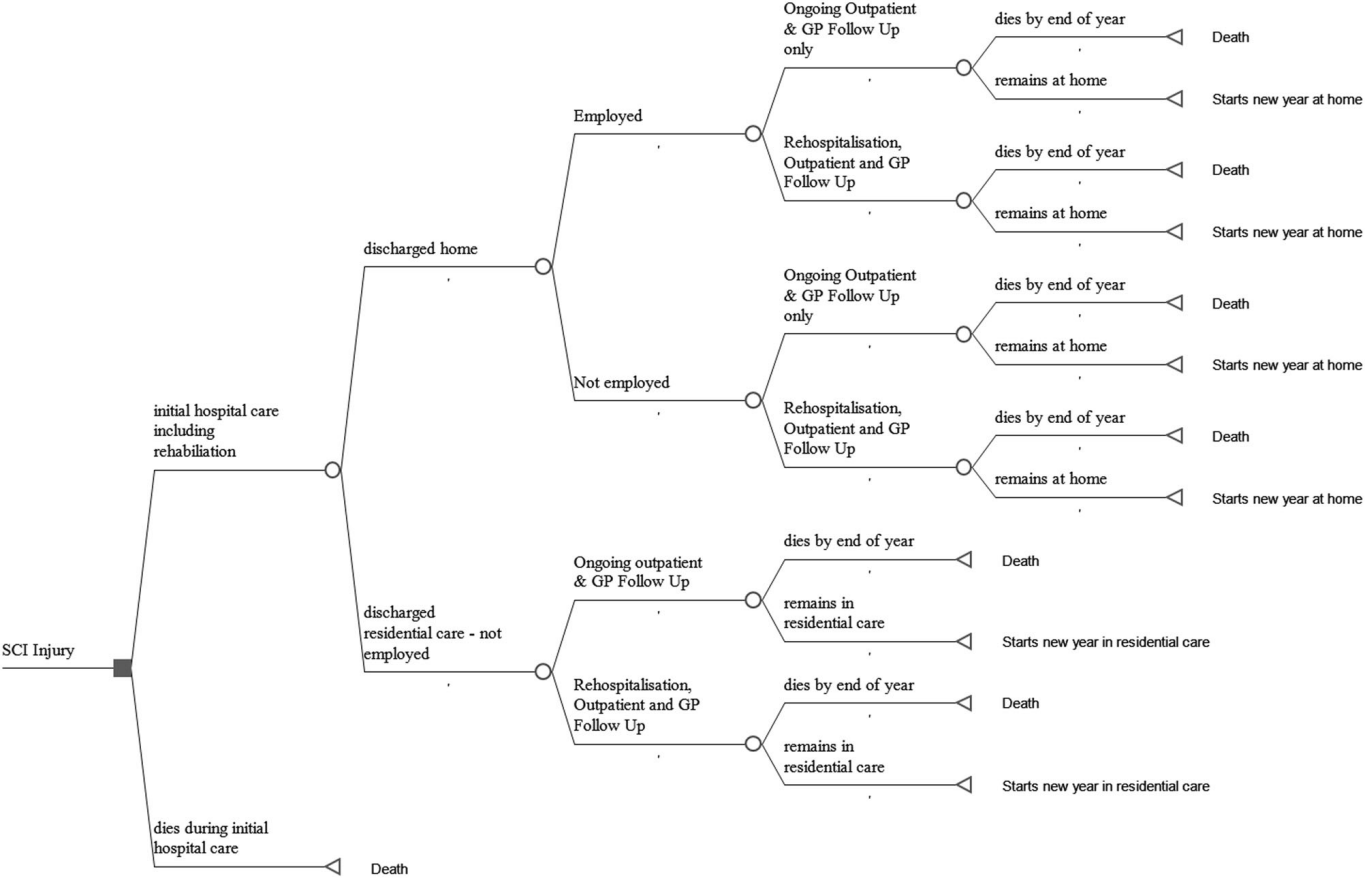
Overview of model structure

A Microsoft-Excel simulation model estimated net present value lifetime costs for all new hospital presenting cases of SCIs in the UK in 1 year. This incidence-based costing approach is powerful as it allows subsequent economic evaluations to illustrate potentially avoidable long-term costs if interventions are effective in preventing and/or reducing the impacts of any health problem [6]. It has been used to estimate lifetime costs of SCIs in Australia, Canada and the US [7–11]. Figure 1 provides an overview of model structure following presentation to hospital for SCIs. If individuals survive hospitalisation, including rehabilitation, they are either discharged home or to residential care. Costs are incurred over remaining lifespans. The economic analysis includes immediate and ongoing healthcare costs directly attributable to SCIs, as well as costs of aids, adaptations, home modifications and provision of home-based and residential care. Unpaid informal care costs and reduced rates of participation in employment are also included.

All costs are in 2016 UK pounds, where necessary using the International Monetary Fund’s World Economic Outlook Purchasing Power Parity 2016 implied inflation and conversion factors. Costs beyond 1 year are discounted at the UK HM Treasury Green Book recommended rate of 3.5% per annum.

### Incidence data

Given the absence of a single source of UK-wide incidence data, the model used data from the Queen Elizabeth National Spinal Injuries Unit (QENSIU). QENSIU (https://www.spinalunit.scot.nhs.uk/) has treated all new cases of traumatic SCI in patients aged >12 years in Scotland for >20 years [1]. Mean annual incidence rates for traumatic and non-traumatic paraplegic and tetraplegic SCIs broken down by gender and 10-year age groups for the period 2009–2013 were available [1].

In QENSIU, neurological level of injury was assessed by a spinal injury consultant on admission and defined according to the International Neurological Classification of Spinal Injury using the American Spinal Injury Association Impairment Scale (AIS). This economic analysis uses this data to group traumatic injuries at admission between more severe AIS-ABC grades for both paraplegia and tetraplegia and all AIS-D grade injuries. Conservatively, it assumes that all reported non-traumatic injuries are AIS-D grade even though at least a third of these injuries could be more severe and costly [12]. UK Office of National Statistics mid-year age- and gender-specific population age estimates for the four UK nations in 2016 were used to calculate the number of new SCIs nationwide.

Many different approaches have been used to estimate short- and long-term impacts on mortality of SCIs [13]. Here costs were adjusted to account for 1-year post-injury mortality rates for paraplegic/tetraplegic AIS-ABC or all D grades [14]. The model accounts for longer-term survival by age group, gender and injury severity using a 70-year study of life expectancy for patients at two English SCI centres [15].

### Resource use and unit costs

Table 1 provides resource use, unit costs and other parameters used in the model. Where possible, we made use of UK sources but otherwise drew on international literature. We used HRG (Healthcare Resource Groups) tariffs for costs of immediate hospital episodes for SCI in the 2015–2016 English National Schedule of Reference Costs [16]. As England represented 84% of the UK population, we applied these costs UK wide, even though they are only used for reimbursement in England. This approach is conservative as some costs in specialist SCI centres may be higher than these tariffs. Tariffs do not directly distinguish between traumatic and non-traumatic injuries; we assume all traumatic SCI events involve an immediate non-elective hospital stay, plus costs for ambulatory transport/treatment. Non-traumatic SCIs conservatively are assumed to involve an episode of elective hospital treatment. Costs of excess bed days beyond the maximum permitted using these tariff codes have been included. We assumed tetraplegic injuries involve the HC21D tariff for the higher level of complications and comorbidities (CC) Score 2+: maximum length of stay of 123 days, beyond which additional payments are made. Conservatively all AIS-D grade injuries are valued using a short-stay tariff used for patients with length of stay of 2 days maximum.

**Table 1:** Parameters used in model

Description of model parameter	Value	Unit	Source
Non-elective spinal cord injury with CC Score 2+ (HC21D)	£20,448.28	Episode (including excess days)	[16]
Elective spinal cord injury with CC Score 2+ (HC21D)	£10,034.00	Episode (including excess days)	[16]
Non-elective spinal cord injury with CC Score 0–1 (HC21E)	£6741.14	Episode (including excess days)	[16]
Elective spinal cord injury with CC score 0–1 (HC21E)	£4305.00	Episode (including excess days)	[16]
Non-elective spinal cord injury with CC Score 2+ (short stay) (HC21D)	£2200.98	Episode	[16]
Non-elective spinal cord injury with CC Score 0–1 (short stay) (HC21E)	£963.17	Episode	[16]
Ambulance see, treat and convey (ASS02)	£236	Per contact	[16]
Non-admitted, face to face consultant led follow- up (WF01A)	£289	Per consultation	[16]
GP consultation	£45	Per consultation	[26]
Tetraplegia ABC year 1 modifications/adaptations	£6781	Per person per annum	[7]
Tetraplegia ABC year 2 modifications/adaptations	£27,144	Per person per annum	[7]
Tetraplegia ABC year 3 modifications/adaptations	£11,935	Per person per annum	[7]
Tetraplegia ABC year 4 modifications/adaptations	£6078	Per person per annum	[7]
Tetraplegia ABC year 5 modifications/adaptations	£5876	Per person per annum	[7]
Tetraplegia ABC year 6 modifications/adaptations	£6527	Per person per annum	[7]
Tetraplegia ABC year 7+ modifications/adaptations	£3263	Per person per annum	[7]
Paraplegia ABC year 1 modifications/adaptations	£5878	Per person per annum	[7]
Paraplegia ABC year 2 modifications/adaptations	£17,178	Per person per annum	[7]
Paraplegia ABC year 3 modifications/adaptations	£16,628	Per person per annum	[7]
Paraplegia ABC year 4 modifications/adaptations	£8578	Per person per annum	[7]
Paraplegia ABC year 5 modifications/adaptations	£8045	Per person per annum	[7]
Paraplegia ABC year 6 modifications/adaptations	£5844	Per person per annum	[7]
Paraplegia ABC year 7+ modifications/adaptations	£2922	Per person per annum	[7]
Tetraplegia ABC rehospitalisation rates in 12 months	44.9	%	[19]
Intermediate specialist nursing home care	£1294	Per week	[23]
High specialist nursing home care	£1656	Per week	[23]
Very high specialist nursing home care	£2277	Per week	[23]
Tetraplegia ABC rehospitalisation rates in 12 months	44.9	%	[19]
Paraplegia ABC rehospitalisation rates in 12 months	35.3	%	[19]
All D grades rehospitalisation rates in 12 months	23.8	%	[19]
Tetraplegia ABC average number of rehospitalisations in 12 months	1.45	Mean rehospitalisations	[19]
Paraplegia ABC average number of rehospitalisations in 12 months	1.28	Mean rehospitalisations	[19]
All D average number of rehospitalisations in 12 months	1.33	Mean rehospitalisations	[19]
Mean number of outpatient consultations per annum	1.79	Mean outpatient consultation per person	[17]
Mean GP consultations per annum	9.94	Mean GP consultations per person	[17]
Discharge rate to nursing homes in 1 year postinjury for all SCI injuries	6	%	[22]
Paraplegia ABC mean hours of professional home care	30.9	Mean hours per week	[25]
Tetraplegia ABC mean hours of professional home care	58.1	Mean hours per week	[25]
All D mean number of hours of professional home care	5	Mean hours per week	Authors’ assumption
Paraplegia ABC mean hours of family home care	16.03	Mean hours per week	[7]
Tetraplegia ABC mean hours of family home care	28.0	Mean hours per week	[7]
All D mean hours of family home care	4.9	Mean hours per week	[24]
Home care (local authority provided)	£30.75	Rate per hour	[26]
Employment rate for paraplegia ABC	40.2	%	[28]
Employment rate for tetraplegia ABC	31.4	%	[29]
Employment rate for all D injuries	71.0	%	[31]
General employment rate in population without disability in the UK	79.0	%	[29]
Minimum wage over 25s	£7.20	Per hour	[27]
Annual wage 16 and 17 year olds	£6386	Per annum	[27]
Median annual wage 18–21	M: £11,283 W: £7695	Per annum	[32]
Median annual wage 22–29	M: £22,221 W: £17,796	Per annum	[32]
Median annual wage 30–39	M: £29,799 W: £20,488	Per annum	[32]
Median annual wage 40–49	M: £33,207 W: £19,319	Per annum	[32]
Median annual wage 50–59	M: £31,842 W: £18,572	Per annum	[32]
Median annual wage 60–67	M: £23,929 W: £12,832	Per annum	[32]

*CC* complications and comorbidities, *GP* general practitioner

Rates of rehospitalisation for discharged rehabilitation patients with SCI can be high, in excess of 50% of patients with more severe trauma may be readmitted at least once within 1 year [17–19]. Common reasons for rehospitalisation include respiratory and urinary tract infections, as well as fractures; this typically occurs in a local hospital rather than specialist SCI centre. Specialist centres deal with highly specialist issues, e.g. pressure sore management, complex rehabilitation needs or spine surgery. Annual readmission rates to any hospital following post-rehabilitation discharge and the number of rehospitalisations were based on US analysis for specialist rehabilitation centres, again distinguishing between rates for tetraplegia and paraplegia ABC grades as well as all D grade injuries [19]. We have very conservatively used short length of stay tariffs for all readmissions, even though some stays will be reimbursed using full tariffs used for initial admissions for ABC grade injuries. All outpatient consultations were valued using a non-mandatory English tariff for consultant-led SCI follow-up consultations, assuming a mean of 1.79 outpatient consultations per annum observed in Denmark [17]. We also drew on this Danish analysis to estimate 9.94 GP consultations per annum related to SCI.

The need for home/vehicle modifications depends on injury severity. A Swiss survey of 482 people with SCI reported 85% had at least one home adaptation [20], most commonly a wheelchair-accessible shower (63%). Published estimates of adaptation costs vary considerably; we use estimates from Australian analysis of SCI costs. This included mean costs for home/transport adaptations, ventilation equipment, special beds and communication aids, as well as vocational equipment, education and training courses [7]. To be conservative, we only applied these costs to the ABC grades of tetraplegia and paraplegia.

The model also accounts for immediate/ongoing costs following discharge to specialist residential care. This is conservative as the increased likelihood of subsequent transfer to residential care after 1 year is not included. Although it is suggested that 20% of UK SCI patients could be discharged to residential care [21], we use a more conservative estimate of 6% reported in discharge data from specialist SCI centres [22]. UK costs for specialist intermediate, high and very high levels of nursing home care are applied to grade D, paraplegia ABC and tetraplegia ABC grades, respectively [23]. In the year postinjury, these costs are adjusted given time already spent in hospital to avoid double counting. Unit costs used are at the low end of reported cost ranges; individuals with more complex needs, e.g. on ventilators, are likely to incur substantially higher costs than used in the model. The model also does not account for higher background risk of residential care admission for older people regardless of SCI status.

The analysis includes costs of long-term home-based attendant/nursing care. This varies depending on care needs, from 24-h 7-day-a-week care to little or no care. An evaluation of specialist neuro-rehabilitation in the north of England simply estimated costs for 168 and 14 h per week care due to lack of data [5]. We identified analysis where 48 patients 1 year after discharge from a specialist SCI centre in England received a mean 80 h of paid care per week, (range 1–168 h) [24]. In all, 63% were classified as having paraplegia or tetraplegia AIS grades ABC with the remainder AIS-D grades at 1 year follow-up.

The model assumes on average individuals with paraplegia or tetraplegia ABC grades living at home receive 30.87 and 58.1 h of paid care per week, respectively. This is more conservative than the 80-h per week estimate in the English analysis we identified. These values are based on a breakdown of care use in the United States [25] that we have reduced to take account of expected informal care received; for all AIS-D grade injuries, we conservatively assume a mean of 5 h of contact per week based on our own expert experience of SCI patients in Scotland. All hours are costed using hourly rates for local authority provided home care in England in 2016 [26].

Few estimates of informal family care provided to people with SCI have been published. We were unable to find UK costs, so we used estimates from Australia on informal care received by individuals with tetraplegia and paraplegia [7]. There is almost no information on informal care for AIS-D grade injuries; the model assumed a mean of 1 h per day based on a small English analysis [24]. As in the Australian analysis, the model assumed only 70% of SCI cases would have access to informal care. Costs were valued using the hourly national minimum wage for the over 25s [27].

Employment rates came from an English survey of 1700 working age people who had used specialist SCI centre care; only 40.2% of paraplegia ABC injuries and 31.4% of tetraplegia ABC injuries were employed [28]. This contrasts with a 79% employment rate for ‘all working age people who are not classified disabled and/or work-limiting disabled’ [29]. Pain, fatigue and older age are among reasons for reduced employment for AIS-Ds, but data on actual employment rates is sparse [30]. We assumed 71% of those of working age would be in employment, using Canadian analysis [31]. Median annual pay rates from the UK Annual Survey of Hours and Earnings are used to value productivity losses for individuals aged 18–67 years [32], with minimum wage rate used for losses in 16–17-year olds [27]. This is conservative; individuals aged <16 years will lose educational opportunities, while some aged >67 years might have remained in the labour market or otherwise contributed to the economy, such as by volunteering or providing care.

## Results

The model estimates 1270 (913 male, 357 female) new SCI cases per annum, of which 13% would be non-traumatic. Table 2 indicates approximately 35% of SCIs would be tetraplegia ABC injuries and 18% paraplegia ABC injuries. Nearly 70% of SCIs are for individuals aged >46 years. Overall estimated lifetime costs for new cases of SCI are £1.43 billion (Table 3). More than £60 million of costs are for the first year of care, with 50% of costs incurred by those aged >56 years (Table 4). In all, 71% of lifetime costs potentially fall on the public purse and 29% of costs are due to reduced employment of people living with SCI, as well family carer time.

**Table 2:** Estimated new cases of SCI in the UK in 2016

Age group, years	Paraplegia—ABC	Tetraplegia—ABC	All D	Total	Percentage of total cases
0–15	3	6	6	16	1.27%
16–25	24	48	59	131	10.31%
26–35	22	43	53	118	9.30%
36–45	22	43	67	132	10.38%
46–55	41	80	116	237	18.67%
56–65	40	79	118	237	18.63%
66–75	44	86	119	249	19.57%
76–85	26	50	58	133	10.50%
86+	4	7	7	17	1.36%
Total	225	442	603	1270	
Percentage of total cases	17.69%	34.80%	47.51%		

*SCI* spinal cord injury

**Table 3:** Lifetime costs of new spinal cord injuries in the UK in 2016 (£s 2016 prices)

All	Paraplegia—ABC	Tetraplegia—ABC	All D	Total cost
Immediate inpatient care costs	1,561,340	9,141,768	1,085,259	11,788,367
Initial home and vehicle modifications	1,259,756	2,680,908	0	3,940,665
Ongoing home modifications	20,018,649	32,834,814	0	52,853,463
Ongoing healthcare costs	5,822,627	14,925,680	18,360,452	39,108,759
Initial home care costs	4,972,049	17,262,371	2,202,335	24,436,755
Ongoing home care costs	179,650,347	511,312,375	93,463,185	784,425,907
Initial residential care costs	553,653	1,404,319	1,183,112	3,141,083
Ongoing residential care costs	20,004,608	41,595,991	50,209,180	111,809,779
Initial family care costs	604,532	1,947,914	505,355	3,057,802
Ongoing family care costs	21,843,002	57,697,312	21,467,517	101,007,831
Productivity losses	59,912,035	135,729,880	96,014,860	291,656,775
Total	316,202,599	826,533,331	284,491,256	1,427,227,187

**Table 4:** Total costs by age group in initial year of SCI in the UK in 2016 (£s 2016 prices)

Age group, years	Paraplegia—ABC	Tetraplegia—ABC	All D	Total cost
0–15	135,574	488,925	63,734	688,232
16–25	1,130,947	3,910,368	758,369	5,799,684
26–35	1,209,899	3,953,977	966,054	6,129,930
36–45	1,245,033	4,033,442	1,268,831	6,547,306
46–55	2,285,565	7,429,094	2,173,540	11,888,199
56–65	2,162,988	7,110,349	2,058,266	11,331,603
66–75	2,312,026	7,648,383	2,007,549	11,967,958
76–85	1,053,393	3,798,898	569,262	5,421,553
86+	145,624	525,169	68,459	739,251
Total cost	11,681,047	38,898,605	9,934,063	60,513,715

*SCI* spinal cord injury

Fifty-one million pounds in specialist hospital-based healthcare service costs is likely to be conservative; these are greatest for tetraplegia ABC cases, accounting for >2.9% of their lifetime costs. This figure does not include further substantive costs in hospital/home for the most complex cases of SCI, including ventilation. Anecdotally, we are aware of annual costs in excess of £320,000 per annum for 24/7 home care for a person with SCI requiring ventilation.

**Fig. 2:**
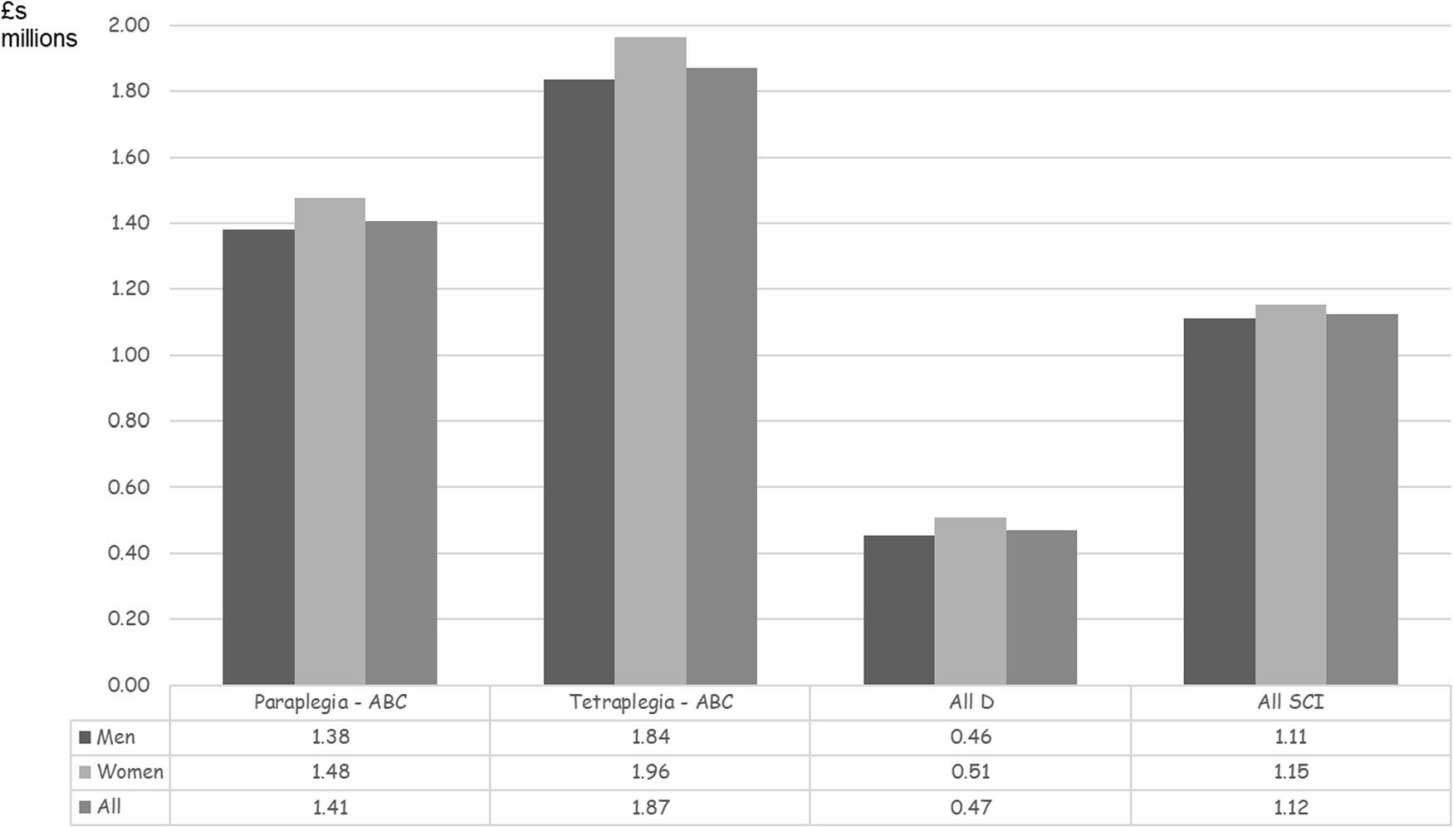
Mean lifetime costs of new spinal cord injuries by type and gender (2016 £ millions)

Figure 2 illustrates mean expected lifetime costs per SCI case. Overall, costs would be £1.12 million per SCI, ranging from £0.47 million per person with grade D injuries to £1.87 million per tetraplegia ABC injury. Mean lifetime costs per woman are higher: £1.15 million versus £1.11 million per man. Median lifetime costs per SCI of £0.72 million are lower reflecting the concentration of injuries in older age groups. Median costs range from £0.40 million per grade D injury to £1.95 million per tetraplegia ABC injury. Median lifetime costs per woman are higher: £0.80 million versus £0.72 million per man.

### Sensitivity analyses

Sensitivity analyses explored impacts of changing key model assumptions. The maximum initial tariff for non-elective traumatic complex spinal injuries used was £20,448, with much lower tariffs used for less severe injuries; even with rapid transfer to specialist SCI centres, care costs for some can be much higher, especially if requiring long-term ventilation. A French study reported 6.5% of new traumatic SCI cases required long-term ventilation [33]. If 6.5% of all traumatic tetraplegia cases required ventilation, with mean 60-day stay in a specialist centre at £981 per day [34] their individual costs would increase to £58,860 and lifetime mean costs for all tetraplegia ABC grades increase from £1.870 million to £1.873 million. Overall costs of SCI would rise marginally to £1.428 billion from £1.427 billion. If 20% of tetraplegia cases required ventilation, costs would rise to £1.431 billion overall and £1.878 million per tetraplegia ABC case. The model thus appears relatively insensitive to increasing immediate healthcare costs associated with the most traumatic cases, although additional costs of complications, such as dealing with pressure sores and infections, are not included. We conservatively used short-stay HRG tariffs for grade D injuries and rehospitalisations for ABC grade injuries. The model is more sensitive to changes in this assumption. For each additional 10% of D injuries and all ABC rehospitalisations that required a longer stay (qualifying full tariff reimbursement), overall costs increased by £13 million.

The model is sensitive to professional home care assumptions. If all individuals with tetraplegia ABC injuries received 7.3 home care hours per day (less informal care time) rather than 12.3 h per day, then overall costs fall to £1.11 billion and mean lifetime costs per individual with tetraplegia ABC injuries to £1.15 million. If total hours of professional care are not reduced to take account of expected mean informal care received, costs increase to £1.78 billion. If the 6.5% of people with tetraplegia ABC who might require long-term ventilation were also to need 24-hour daily home care, then total lifetime costs would rise to £1.83 billion, with mean lifetime costs per tetraplegia ABC case increasing to £2.56 million.

The home care hourly rate also impacts on care costs. If a lower hourly rate for personal care of £14.28 [26] is used, then overall costs of SCIs fall to £1.18 billion. The model is also sensitive to assumptions on use of long-stay residential care. If 20% (rather than 6%) of tetraplegia and paraplegia ABC cases were discharged from hospital to residential care [21], then overall costs increase to £1.80 billion, with mean lifetime costs of £2.48 million and £1.85 million, respectively.

## Discussion

An incidence-based costing approach provides policymakers with information on long-term impacts; new UK cases of SCI per annum may incur lifetime costs exceeding £1.43 billion or £1.12 million per case, with more than two thirds of costs potentially falling on the public purse.

This estimate is conservative. For instance, £21.4 million is spent annually on spinal cord rehabilitation services in England (HRG code VC08Z) [22], but we do not know the proportion for new SCI cases. We have not included additional costs for treatment/management of specific complications or multi-morbidities, including any support from mental health specialists. In Australia, initial hospitalisation costs for those aged >65 years who experienced traumatic SCI were 30% higher (mean difference £3611) than for the under 65s due to greater co-morbidities [35]. Nor does it include lifetime costs of essential drug therapies or ventilator dependence. We omit costs associated with delays in reaching specialist SCI units from critical care settings, as well as avoidable delayed discharges from specialist settings due to limited suitable accommodation [34]. We omit adverse impacts on quality of life or social exclusion that substantially increase societal costs. Our estimate of non-traumatic SCI cases is probably conservative, based on attendance at one specialist SCI centre, as some less severe cases may not be referred to these services. We have not captured costs of treatment activity for non-traumatic SCI related to underlying morbidities, such as vascular disorders or malignancies, where these costs are reimbursed under a different HRG tariff. Furthermore, we assumed a high employment rate for AIS-D cases (71%), but little is known on actual employment rates, nor does our model account for return to part-time rather than full-time work.

International studies point to substantive costs but estimates vary greatly and interpretation is difficult due to differences in health/social care systems. Studies use a variety of methodologies, define SCIs in different ways and focus on different elements of cost. Few include non-traumatic SCIs.

Several used incidence-based costing approaches. A model for traumatic SCIs in Australia reported lifetime costs of £2.73 million and £5.2 million per paraplegia or tetraplegia case, respectively. These costs are higher than our analysis (£1.41 million and £1.87 million, respectively), mainly because the Australian model includes the monetary value for lost quality of life due SCI disability. It estimated that each year of full quality life lost would cost £91,750 [7]. These costs are not borne by the public purse and the values are open to challenge; in the UK values between £20,000 and £30,000 per year of full quality life are typically used [2]. Excluding these costs and looking solely at health, long-term care, aids and adaptations, lifetime costs in the Australian model for paraplegia and tetraplegia were £1.11 million and £2.77 million, respectively. Remaining differences are due to Australian inclusion of some costs of treating multi-morbidities, as well as not accounting for the lower costs of D grade injuries.

A Canadian model estimated lower lifetime costs (including quality-of-life losses valued at £28,841 per year) between a mean £0.89 million for incomplete paraplegia to £1.82 million for complete tetraplegia [8]. This is mainly due to much lower assumed costs for residential care. Another Canadian study reported net lifetime healthcare costs per traumatic SCI case compared to a matched population without SCI. Incremental costs were between £0.14 million and £0.28 million, depending on rehabilitation and pre-existence of pressure ulcers [9]. Gross lifetime costs, not deducting non-SCI healthcare costs, were modestly higher, ranging from £0.17 million to £0.31 million.

In a US model health, rehabilitation and long-term care mean lifetime costs were between £0.58 million and £4.20 million depending on age and injury severity [10]. In another, mean lifetime all-cause hospital costs for thoracic SCI were calculated using data from 14 specialist SCI centres [11]. Costs were £0.23, £0.18, £0.13 and £0.05 million for each AIS grade A–D, respectively. This study did not include health, long-term care or rehabilitation costs outside of hospital.

In our analysis, mean initial year costs per case for hospitalisation, aids and adaptation, as well as any residential or home care use, were £39,846, £73,393 and £8,246 for paraplegia ABC, tetraplegia ABC and AIS-D, respectively. In Australia, mean initial costs of hospitalisation for traumatic SCI, including initial rehabilitation as well as acute care, were estimated as £11,652 and £15,264 for individuals aged up to 64 and aged ≥65 years, respectively [35]. Costs in the Australian analysis varied dramatically depending on type of injury with mean total hospital costs for sub-groups of individuals ranging from £5353 for mild-to-moderate injuries for the younger cohort compared with £19,956 for serious injuries for the older cohort. In  Canadian analyses, mean initial year hospital costs for traumatic SCI cases covering health, rehabilitation and continuing care were £72,496 [9] and £75,192 [36]. Costs for complete SCI of £92,283 and £31,950 for incomplete SCI were also reported [37]. In Spain, first year costs of SCI due to motor vehicle trauma (MVT) or all other causes were modelled [38]. Average healthcare costs, adaptations and specialist ongoing care for the MVT and non-MVT groups were £31,092 and £30,593, respectively.

We conclude that, despite the magnitude of costs, this first analysis of UK SCI costs is conservative. Further analyses of UK costs, making use of registry data on long-term resource use, are needed. This would allow analysis to account for SCIs converting from one ASIA grade to another [39]. Work is also needed worldwide to better understand non-traumatic SCI costs. Our model could be adapted and further developed as these data become available.

**Fig. 3:**
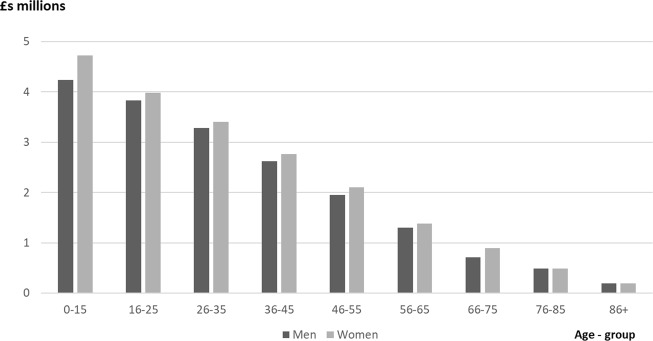
Mean lifetime costs per new tetraplegic traumatic spinal cord injury by age-group and gender (2016 £ millions)

Our analysis shows how models play a powerful role in highlighting economic impacts of SCI to policymakers and illustrating how changes in patterns of cause, type, age at injury and mortality risk influence future costs. This is important given trends towards higher age at injury, for instance linked to falls rather than traffic accidents. In our model, age at injury substantially impacts on costs, for instance with mean lifetime cost of traumatic tetraplegia in a man aged 36–45 years being £2.63 million compared with £1.30 million if aged between 56 and 65 years (Fig. 3), but as Table 4 illustrates 49% of aggregate costs for the first year of treatment are incurred by those aged >56 years. Models are vital tools in bringing together estimates of SCI costs with evidence on effective ways of improving outcomes at individual and population level. They help provide evidence on key policy concerns to be addressed, including the economic case for improved care pathways, prompt referral to specialist SCI centres and reductions in delayed discharges following rehabilitation. Our model also implies a case for evaluating the long-term cost-effectiveness of rehabilitation programmes. If effective in improving compensatory skills and helping SCI patients to use devices that maximise functional performance, then substantive lifetime costs to the public purse and families may be averted.

## Data archiving

The Excel model used to estimate these costs is available on request from the corresponding author. It is also available to download from https://www.spinal-research.org/cost-spinal-cord-injury.
